# Increased Circulating T Follicular Helper Cells Induced *via* IL-12/21 in Patients With Acute on Chronic Hepatitis B Liver Failure

**DOI:** 10.3389/fimmu.2021.641362

**Published:** 2021-03-31

**Authors:** Bingying Du, Jiaming Teng, Rongkun Yin, Yuanyuan Tian, Tingwang Jiang, Yanan Du, Wei Cai

**Affiliations:** ^1^ Department of Infectious Diseases, Ruijin Hospital, Shanghai Jiao Tong University School of Medicine, Shanghai, China; ^2^ Department of Infectious Diseases, Tongren Hospital, Shanghai Jiao Tong University School of Medicine, Shanghai, China; ^3^ Department of Hematology, Children Hospital, Soochow University, Suzhou, China; ^4^ Clinical Research Centre, The Affiliated Changshu Hospital of Xuzhou Medical University, Changshu, China

**Keywords:** T follicular helper cell, hepatitis B virus-related acute on chronic liver failure, pathogenesis, interleukin-21, interleukin-12

## Abstract

**Objectives:**

T Follicular helper (Tfh) cells, recognized as a distinct CD4^+^ T cell subset, mediate the development of long-lived humoral immunity *via* B cell activation/differentiation. Tfh cells play an important role during hepatic viral infection, but its role in hepatitis B virus-related acute on chronic liver failure (HBV-ACLF) remains to be explored.

**Materials and Methods:**

The frequency of Tfh cells, serum pro-inflammatory cytokine (IL-12, IL-21, IL-17 and TNF) levels and IgG/M levels were investigated in HBV-ACLF (n = 36), serious chronic hepatitis B (n = 21), moderate chronic hepatitis B patients (n = 32) and healthy control (HC) subjects (n = 10).

**Results:**

Circulating Tfh cells were significantly increased in HBV-ACLF patients compared to other groups, correlating well with MELD score. However, the frequency of Tfh cells decreased in ameliorated HBV-ACLF patients. Furthermore, serum IL-12 and IL-21 levels were higher in HBV-ACLF patients, compared to other groups. Naïve CD4^+^ T cells from HC subjects differentiate into Tfh cells following treatment with HBV-ACLF patients’ serum, a process that can be blocked by IL-12/21 neutralizing antibodies. Tfh cells induced by HBV-ACLF patient’s serum promoted the proliferation and IgG production of B cells *in vitro*. Moreover, circulating CD19^+^ B cells, serum and liver IgG/M levels were significantly higher in HBV-ACLF patients, compared to other groups.

**Conclusions:**

Our data demonstrated that there was a high frequency of Tfh cells and high levels of serum IL-12/21 in HBV-ACLF patients. Naïve CD4^+^ T cells differentiate into Tfh cells in the presence of HBV-ACLF patients’ serum rich in IL-12/21, which can be blocked by neutralizing IL-12/21 antibodies. These data may provide useful insights for both clinical and basic research in the treatment of HBV-ACLF.

## Introduction

Hepatitis B virus-related acute on chronic liver failure (HBV-ACLF) has recently been characterized as an acute hepatic insult, manifesting as jaundice and coagulopathy, complicated within 4 weeks by ascites and/or encephalopathy in a patient with previously chronic hepatitis B virus (HBV) infection (1). Due to the extremely poor prognosis of HBV-ACLF, liver transplantation remains the most effective therapy (1), limited by the shortage of suitable donors. Therefore, it is critically important to understand the underlying molecular mechanism of HBV-ACLF to facilitate the development of therapeutic target(s).

It has been reported that HBV-ACLF is associated with Th1 and Th17 for cell mediated (2, 3) and humoral immunity (4). It is well known that CD4^+^ T cells differentiate into Th1, Th2, Th17, and Treg cells. Recently, a new class of T cells, specializing in promoting B cell differentiation in the lymphoid follicles, was characterized, and named T follicular helper (Tfh) cells (5). Tfh cells can be distinguished from other CD4^+^ T cell lineages by their expression of a unique combination of effector molecules, including high levels of CXCR5, ICOS, PD-1, IL-21 and Bcl-6 (6, 7). Circulating CD4^+^CXCR5^+^ T cells have been found in recent studies to have functional characteristics similar to Tfh. Indeed, circulating CD4^+^CXCR5^+^ T cells promote survival, proliferation and differentiation of B cells into plasma cells as Tfh cells are present in germinal centers. However, the phenotype of circulating Tfh differs from “conventional” tissue-specific Tfh cells that express high levels of PD-1 and ICOS. Thus, the definition of circulating Tfh cells differs in various studies (8, 9).

IL-12, produced by dendritic cells, macrophages and B cells in response to microbial pathogens (10), is important in differentiation and generation of Tfh cells *via* the induction of IL-21 and Bcl-6 genes (7). Tfh cells produce higher amounts of IL-21 than Th1 and Th2 subsets (11). IL-21, a genuine T cell co-stimulator, is important in lymphocyte activation, survival, and differentiation (12). IL-21 is up-regulated in HIV and HBV infection and plays a vital role in the control of chronic viral infection (13, 14), correlating with increased circulating Tfh cells (15). However, the role of Tfh cells in the pathogenesis of HBV-ACLF remains unclear.

Thus, the frequency of Tfh cells and IL-12/21 levels in HBV-ACLF, chronic hepatitis B (CHB) and healthy controls (HC) subjects were investigated in the current study to gain insight into the role of Tfh cells in patients who developed HBV-ACLF.

## Materials and Methods

### Human Subjects

A total of 99 subjects were recruited in the *Ruijin Hospital, Shanghai Jiao Tong University School of Medicine*, including HBV-ACLF (n = 36), severe CHB (S-CHB) (n = 21), moderate CHB (M-CHB) patients (n = 32), and healthy controls (HC) subjects (n = 10). Main clinical data of these subjects were shown in **Table 1**. All patients had positive hepatitis B surface antigen for more than 6 months. S-CHB was defined as elevation of alanine aminotransferase (ALT) ≥ 10-fold upper limit of normal (ULN, 64 IU/L), total bilirubin (TBIL) ≥ 5-fold ULN (17.1 µmol/L). M-CHB was defined as elevation of ALT ≥ 3-fold ULN and < 10-fold ULN, TBIL ≥ 2-fold ULN and < 5-fold ULN. HBV-ACLF patients, according to a recent Asia-Pacific consensus recommendation (1), had a history of CHB with TBIL ≥ 10-fold ULN, prothrombin activity (PTA) < 40%, within 4 weeks by hepatic encephalopathy (≥ grade 2), plus ascites or hepatorenal syndrome. All patients received antiviral therapy (entecavir). Seven of 36 HBV-ACLF patients ameliorated with within 3 months. The patients co-infected with any other virus (hepatitis A, C, D or E) were excluded. This study was conducted according to the ethical guidelines of the 1975 Declaration of Helsinki and approved by the *Ethics Committee of Ruijin Hospital, Shanghai Jiao Tong University School of Medicine*. Informed consent in writing was obtained from each subject.

**Table 1:** The demographic and clinical characteristics of subjects.

	HC (n=10)	M-CHB (n=32)	S-CHB (n=21)	HBV-ACLF (n=36)	*p*
Age, years	48 (22-61)	41 (24-71)	36 (20-66)	44.5 (24-76)	0.255
Sex, male/female (n)	8/2	28/4	20/1	31/5	0.633
ALT, U/L	26.0 (17-38)	223.0 (24-609)	908.0 (35-2206)	148.5 (25-1227)	<0.001
AST, U/L	23.5 (20-32)	69.5 (23-326)	426.0 (37-1960)	173.0 (37-1290)	<0.001
TBIL, µM	12.5 (7.3-18.3)	26.7 (11.1-79.5)	122.8 (15.9-404.0)	478.7 (220.6-724.8)	<0.001
Albumin, g/L	44.0 (39-50)	36.0 (29-46)	36.0 (21-50)	28.5 (18-41)	<0.001
Creatinine, µM	NA	67.5 (43-109)	73.0 (41-97)	86.0 (42-1017)	0.010
PTA, %	NA	96.30 (46-149)	72.22 (45-124)	27.19 (11-39)	<0.001
INR	NA	1.11 (0.96-1.58)	1.26 (1.02-1.60)	2.20 (1.74-4.46)	<0.001
HBV DNA, log_10_ copies/mL	NA	6.35 (2.70-8.00)	5.60 (3.02-8.00)	5.98 (2.81-8.00)	0.693
HBeAg, Pos/Neg (n)	NA	19/13	11/10	17/19	0.230
MELD score	NA	8 (2-14)	14 (4-19)	27 (20-49)	<0.001

All values are expressed as median (range).

HC, healthy controls; M-CHB, moderate chronic hepatitis B; S-CHB, severe chronic hepatitis B; HBV-ACLF, Hepatitis B virus-related acute on chronic liver failure; ALT, alanine aminotransferase; AST, aspartate aminotransferase, TBIL, total bilirubin; PTA, prothrombin activity; HBV, hepatitis B virus; NA, not available.

### Processing of Blood, Liver Tissue Samples, Cell Isolation and Purification

Peripheral blood mononuclear cells (PBMCs) were isolated by density-gradient centrifugation over Ficoll-Hypaque solution from all participants for flow cytometric analysis, as previously described (16). Serum was obtained from every participant for cytokine and immunoglobulin (Ig) analysis.

Liver tissues were obtained from HBV-ACLF patients (n = 6) and HC subjects (n = 4) for histopathology and immunohistochemistry analysis.

Naïve CD4^+^ T cells from HC subjects were purified from PBMCs using a cocktail of biotin-conjugated anti-human monoclonal antibodies, and anti-biotin microbeads (Miltenyi Biotec GmbH, Bergisch Gladbach, Germany), following manufacturers’ instructions.

### Flow Cytometric Analysis

The PBMCs were stained with PerCP-anti-CXCR5 (Biolegend, San Diego, USA), APC-anti-CD4, FITC-anti-ICOS, PEcy7-anti-PD-1, FITC-anti-CD19, PE-anti-IL-21 (ebioscience, San Diego, USA) after washing, as per manufacturer’s instructions. Isotype-matched control IgG was used in all procedures. For IL-21 staining, PBMCs were stimulated with Cell Stimulation Cocktail (ebioscience, San Diego, USA) at 37°C for 6 hours. Then the cells were harvested and stained with the above antibodies. The stained cells were then analyzed, using Becton Dickinson FACSCan and FlowJo software.

### ELISA

Serum levels of IL-1β, IL-2, IL-4, IL-6, IL-8, IL-10, IL-12p70, TNF, IFN-γ, TGF-β (R&D systems, USA), IL-17, IL-21 (PeproTech, USA), IgG and IgM (BeckmanCoulter, Germany) were performed using ELISA kits as per manufacturer’s instructions. The levels of IL-21 and IgG in culture supernatants were detected by ELISA kits.

### Histopathology Staining

The liver tissues were sectioned at 4 µm for histopathology staining, as previously described (17). For immunohistochemistry (IHC), paraffin sections were incubated with antibodies against IgM (Abcam, #ab134159), IgG (Abcam, #ab109489) following previous work (16). For immunofluorescent (IF), paraffin sections were incubated with antibodies against rabbit anti-human CXCR5 (Abcam, #ab133706) and goat anti-human CD4 (R&D systems, #AF-379-NA), followed by Alexa Fluor 488 or 555 conjugated secondary antibodies (Invitrogen, Carlsbad, CA). The images were digitized using a Zeiss fluorescence microscope (LSM 710).

### Stimulation of Naïve CD4+ T Cells *via* Cytokines IL-12, IL-21 or IL-17

Naïve CD4^+^ T cells (1 ×10^5^ cell/well) from HC subjects (n = 6) were stimulated with dynabeads^®^ human T-activator CD3/CD28 (Invitrogen, USA) in flat-bottom 96-well plates, and cultured in the presence of IL-12 (PeproTech, USA) (10 ng/ml), IL-21 (PeproTech, USA) (20 ng/ml), IL-12 + IL-21, or IL-17 (PeproTech, USA) (10 ng/ml) for 72 hours, respectively.

### Stimulation of Naïve CD4+ T Cells With Serum From HBV-ACLF Patients and/or Neutralizing IL-12, IL-21 or IL17 Antibody

Naïve CD4+ T cells (1 × 10^5^ cell/well) from HC subjects, stimulated with dynabeads^®^ human T-activator CD3/CD28, as described above, were cultured in RPMI complete medium with different concentrations of HBV-ACLF patients’ serum (n = 7) and HC subjects’ serum (n = 6). For blocking assays, neutralizing antibody to IL-12 (1 µg/ml, PeproTech, USA), IL-21 (1 µg/ml, PeproTech, USA), IL-12 + IL-21 or IL-17 (1 µg/ml, PeproTech, USA) was added into the mixed culture medium for 72 hours, respectively.

### Cultures of B and T Cells

The sorting was conducted using BD FACS ARIA II (BD Biosciences, San Diego, CA, USA) to acquire naïve B cells (defined as CD27-IgD+CD19+ cells) from HC subjects’ PBMCs. Naïve CD4^+^ T cells (1 ×10^5^ cell cells each/well) after stimulating with the RPMI complete medium, dynabeads^®^ human T-activator CD3/CD28 or HBV-ACLF patients’ serum were cultured with 2 ×10^5^ naïve B cells (defined as CD27^-^IgD^+^CD19^+^ cells) in the presence of a surperantigen (Cytostim, human, Miltenyi Biotec) in RPMI-1640 with 10% heat-inactivated fatal bovine serum. The proliferation of B cells was evaluated using a CFSE dilution assay.

### Statistical Analysis

Differences were evaluated using SPSS 21.0 (Chicago, IL, USA). Continuous variables were expressed as median (range). The multiple clinical characteristics of subjects were compared by Kruskal-Wallis tests, except the age and status of hepatitis B e antigen (HBeAg) were assessed by Chi square test. The data were compared using Mann-Whitney U test, which were not normally distributed between different groups. Interclass comparisons of the same group were made with Wilcoxon’s signed-rank test. Statistical associations were assessed with the Spearman rank order correlation coefficient. And *p* < 0.05 (two-sided) was considered to be statistically significant.

## Results

### Increased Frequency of Tfh Cells in HBV-ACLF Patients

**Figure 1:**
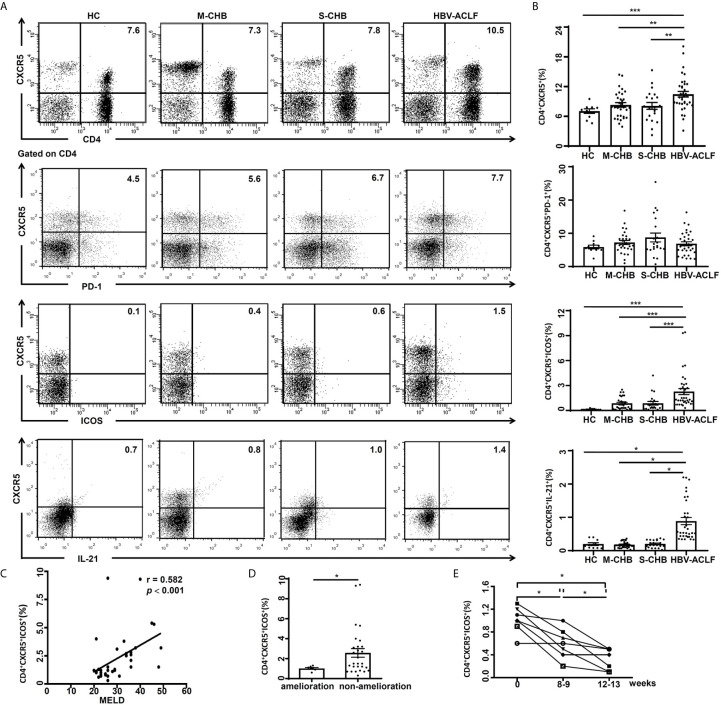
High frequency of Tfh cells in HBV-ACLF patients was associated with disease severity. **(A)** The frequencies of CD4^+^CXCR5^+^, CD4^+^CXCR5^+^ICOS^+^ and CD4^+^CXCR5^+^IL-21^+^ Tfh cells in the PBMCs from HBV-ACLF (n = 36), M-CHB (n = 21), S-CHB (n = 32) patients and HC (n = 10) subjects were demonstrated by flow cytometry. **(B)** The frequencies of CD4^+^CXCR5^+^, CD4^+^CXCR5^+^ICOS^+^ and CD4^+^CXCR5^+^IL-21^+^ Tfh cells in the PBMCs from HBV-ACLF, M-CHB, S-CHB and HC subjects were analyzed using Mann-Whitney U test. **(C)** The correlation between frequency of CD4^+^CXCR5^+^ICOS^+^ Tfh cells and MELD score was analyzed using Spearman correlation analysis. **(D)** The frequency of CD4^+^CXCR5^+^ICOS^+^ Tfh cells from ameliorated (n = 7) and non-ameliorated patients (n = 29) were analyzed by flow cytometry**. (E)** The frequencies of Tfh cells from ameliorated patients (n = 7) over 8- and 12-week treatment were analyzed by flow cytometry. Interclass comparison was made using Wilcoxon’s signed-rank test. *p < 0.05, **p < 0.01, ***p < 0.001.

Peripheral blood was collected from HBV-ACLF (n = 36), S-CHB (n = 21), M-CHB (n = 32) and HC subjects (n = 10) subjects. PBMCs were gated on CD4^+^ T lymphocyte cells and identified CD4^+^CXCR5^+^, CD4^+^CXCR5^+^PD-1^+^, and CD4^+^CXCR5^+^ICOS^+^ Tfh cell subsets in the peripheral blood. The frequency of CD4^+^CXCR5^+^ Tfh cells was up-regulated by 1.3, 1.3 and 1.4-fold in HBV-ACLF patients, compared to S-CHB (*p* < 0.001), M-CHB (*p* < 0.001) and HC subjects (*p* < 0.001); whereas there was no significant difference in the frequency of CD4^+^CXCR5^+^PD-1^+^ Tfh cells among HBV-ACLF and other groups (**Supplementary Figures 1B, C**). An increased frequency of CD4^+^CXCR5^+^ICOS^+^ Tfh cells (**Figures 1A, B**) was also detected in HBV-ACLF patients, which was 2.6, 2.4 and 13.0-fold higher than that in S-CHB (*p* < 0.001), M-CHB (*p* < 0.001) and HC subjects (*p* < 0.001), respectively (**Figures 1A, B**). Furthermore, there were no statistical differences in the frequency of CD4^+^CXCR5^-^ICOS^+^ T cells among groups (**Supplementary Figures 1B, C**). And our further study showed that the frequency of CD4^+^CXCR5^+^ICOS^+^PD-1^+^ Tfh cells in HBV-ACLF patients was 1.7, 2.4 and 6.7-fold higher than that in S-CHB (*p* < 0.05), M-CHB (*p* < 0.01) patients and HC (*p* < 0.01) subjects, respectively(**Supplementary Figures 1B, C**). The activated Tfh cells, which produced IL-21, induced B cell differentiating into antibody-producing cells. And our further study showed that the frequency of CD4^+^CXCR5^+^IL-21^+^ Tfh cells in HBV-ACLF patients was 4.7, 5.1 and 4.5-fold higher than that in S-CHB (*p* < 0.05), M-CHB (*p* < 0.05) patients and HC (*p* < 0.05) subjects, respectively(**Figures 1A, B**).

### Association Between the Frequency of Circulating CD4+CXCR5+ICOS+ Tfh Cells and Disease Severity, Amelioration After Treatment in HBV-ACLF Patients

Model for end stage liver disease (MELD) score is a well-established quantification for prospectively developed and validated chronic liver disease severity (18). In patients with chronic liver disease, there is an association of MELD score and severity of hepatic dysfunction/mortality (18). In the current study, the frequency of CD4^+^CXCR5^+^ICOS^+^ Tfh cells was significantly correlated with MELD score (*r* = 0.584, *p* < 0.001) in HBV-ACLF patients (n = 36) (**Figure 1C**), but not with levels of ALT, AST or HBV DNA.

We also investigated the relationship between the frequency of Tfh cells and amelioration in HBV-ACLF patients. The frequency of Tfh cells from the ameliorated patients (n = 7) was reduced > 50% (*p* < 0.05) compared to non-ameliorated HBV-ACLF patients (n = 29) (**Figure 1D**). We further investigated the relationship between the frequency of Tfh cells and amelioration of HBV-ACLF following treatment. The frequency of Tfh cells was reduced by 40% at 8 weeks (*p* < 0.05) or 60% at 12 weeks (*p* < 0.05) with nucleoside analogue treatment in ameliorated patients, compared to pre-treatment (**Figure 1E**). This data suggests that the frequency of CD4^+^CXCR5^+^ICOS^+^ Tfh cells is a marker for the stage and the severity of HBV-ACLF.

### Increased Levels of Circulating B Cells/Ig and Liver Tfh Cells in HBV-ACLF Patients

**Figure 2:**
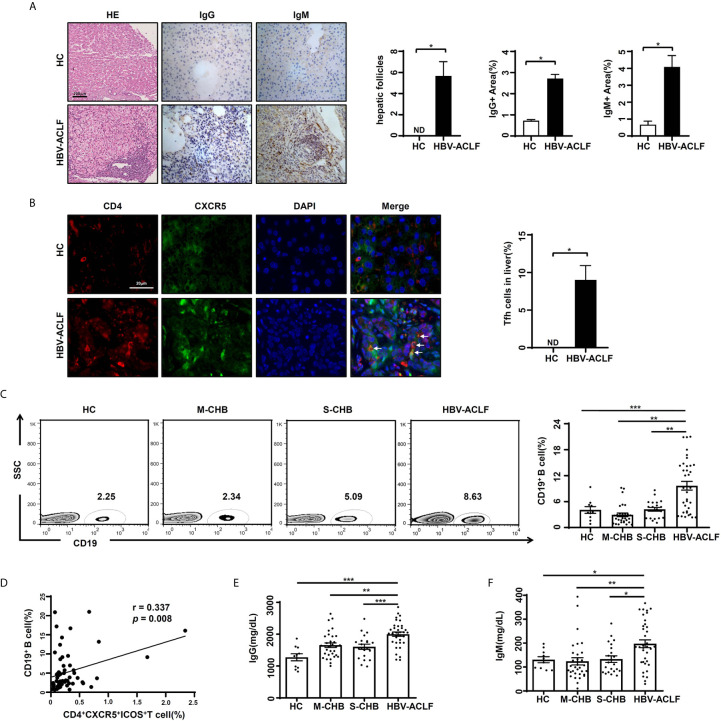
Increased levels of circulating B cells/Ig and liver Tfh cells in HBV-ACLF patients **(A)** Representative hematoxylin-eosin and IHC images of hepatic IgG and IgM expression in HBV-ACLF patients and HC subjects. **(B)** Representative IF images of hepatic Tfh cells in HBV-ACLF patients and HC subjects. **(C)** The frequency of CD19^+^ B cells in the PBMCs from HBV-ACLF (n = 36), M-CHB (n = 21), S-CHB (n = 32) patients and HC (n = 10) subjects were demonstrated by flow cytometry. **(D)** The correlation between frequency of CD19^+^ B cells and CD4^+^CXCR5^+^ICOS^+^ Tfh cells was analyzed using Spearman correlation analysis. **(E, F)** The serum levels of IgG and IgM were detected from HBV-ACLF patients (n = 36), S-CHB (n = 21), M-CHB (n = 32) patients and HC (n = 10) subjects by ELISA. *p* values are tested for the significance of comparisons using Man-Whitney U test. *p < 0.05, **p < 0.01, ***p < 0.001.

Follicle-like structure formation is a fundamental feature of Tfh cells following lymphoid infiltration. The function of Tfh cells is inducing B cells to terminally differentiate for specific antibody production (5). The follicle-like structure in the liver was observed (**Figure 2A**) from HBV-ACLF patients (n = 6), but not from HC subjects (n = 4) (*p* < 0.05). The expression of IgG and IgM in the liver from HBV-ACLF patients was significantly increased (**Figure 2A**), compared with HC subjects (*p* < 0.05). More importantly, the signals of double-positive for CD4 (red)/CXCR5 (green) that presented for Tfh cells were found in the liver tissues from HBV-ACLF patients (**Figure 2B**). These data showed that Tfh cells infiltrated into liver tissue.

To identify the relationship between Tfh cells and B cells, we detected the frequency of circulating CD19^+^ B cells. The frequency of CD19^+^ B cells in HBV-ACLF patients was 2.4, 3.4 and 2.5-fold higher than that in S-CHB (*p* < 0.001), M-CHB (*p* < 0.01) and HC subjects (*p* < 0.01), respectively (**Figure 2C**). Meanwhile, there was a significant correlation between the frequency of circulating CD4^+^CXCR5^+^ICOS^+^ Tfh cells and CD19^+^ B cells (*r* = 0.337, *p* < 0.01) in HBV-ACLF patients (**Figure 2D**). This is supported by the significant up-regulated serum level of IgG (**Figure 2E**) in HBV-ACLF patients compared with S-CHB (*p* < 0.001), M-CHB (*p* < 0.01) and HC subjects (*p* < 0.001), which were 1.4, 1.3 and 1.6-fold higher, respectively. Similar pattern of IgM (**Figure 2F**) was also observed in HBV-ACLF patients, which was 1.6, 1.7 and 1.4-fold significantly higher than that in S-CHB (*p* < 0.05), M-CHB (*p* < 0.01), and HC subjects (*p* < 0.05). These data suggest the close relationship between Tfh cells and B cells in HBV-ACLF.

### Evaluation of Serum Cytokines in HBV-ACLF Patients

**Figure 3:**
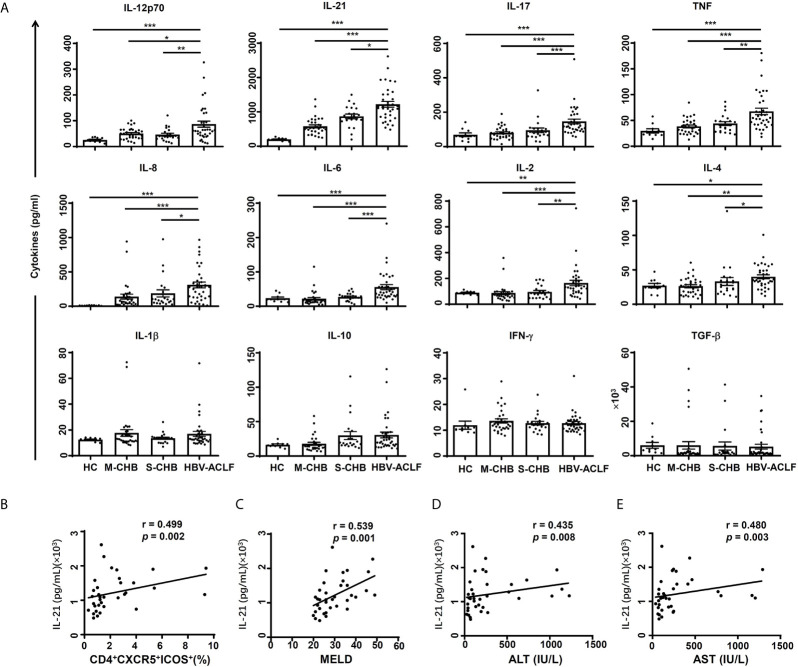
Concentrations of serum cytokines were compared among groups. **(A)** The levels of cytokines (IL-12p70, IL-21, IL-17, TNF, IL-8, IL-6, IL-2, IL-4, IL-1β, IL-10, IFN-γ and TGFβ) in serum from HBV-ACLF (n = 36), S-CHB (n = 21), M-CHB (n = 32) patients and HC (n = 10) subjects were detected by ELISA. *p* values are tested for the significance of comparisons using Man-Whitney U test. **(B–E)** The correlations of IL-21 level with the frequency of CD4^+^CXCR5^+^ICOS^+^ Tfh cells **(B)**, MELD score **(C)**, levels of ALT **(D)** and AST **(E)** in HBV-ACLF patients. *r*, the spearman rank order correlation coefficient. *p < 0.05, **p < 0.01, ***p < 0.001.

The level of serum IL-21 from HBV-ACLF patients was up-regulated by 1.3, 2.2 and 5.8-fold in HBV-ACLF patients, compared to S-CHB (*p* < 0.05), M-CHB (*p* < 0.001) and HC subjects (*p* < 0.001) (**Figure 3A**); whereas levels of IL-12 and IL-17 were up-regulated by ~2.0-fold in HBV-ACLF patients compared to that in other three groups (**Figure 3A**). Similar patterns of IL-2, IL-4, IL-6, IL-8 and TNF (**Figure 3A**) were detected in HBV-ACLF patients and other three groups. No significant differences in IFN-γ, IL-1β, IL-10, or TGF-β were observed among all of the groups.

### Correlation Between IL-21/IL-12 and CD4+CXCR5+ICOS+Tfh Cells or Disease Severity in HBV-ACLF Patients

The correlation between IL-21 and severity of HBV-ACLF was investigated as other studies showed IL-21 has a critical role in Tfh proliferation/differentiation in HIV and Sjögren’s syndrome (15, 19). IL-21 level was significantly correlated with the frequency of CD4^+^CXCR5^+^ICOS^+^ Tfh cells (*r* = 0.499, *p* < 0.01) (**Figure 3B**) and MELD score (*r* = 0.539 *p* < 0.01) (**Figure 3C**), ALT level (*r* = 0.435, *p* < 0.01) (**Figure 3D**) and AST level (*r* = 0.480, *p* < 0.01) (**Figure 3E**) in HBV-ACLF patients (n = 36). However, IL-21 level was not correlated with HBV DNA level (data not shown). In contrast, there was no significant correlation between IL-12 level and the frequency of CD4^+^CXCR5^+^ICOS^+^ Tfh cells, MELD score, ALT or AST levels in HBV-ACLF patients (data not shown). Further, no significant correlation between IL-17 level and the frequency of Tfh cells or MELD scores was observed (data not shown). These findings suggest that the measurement of IL-21 might lead to a simple data-driven test for predicting the development of HBV-ACLF disease.

### Tfh Cells Induction by Soluble Factor in the Serum of HBV-ACLF Patients

**Figure 4:**
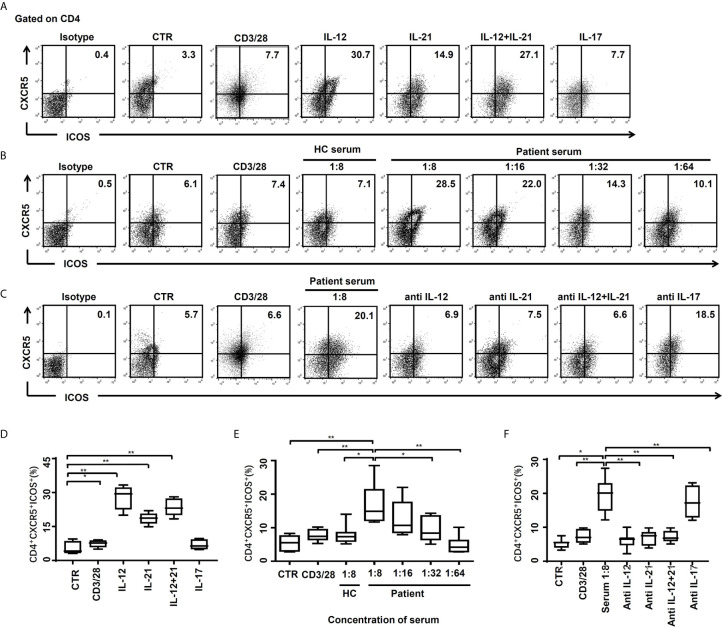
CD4^+^CXCR5^+^ICOS^+^ Tfh cells were induced by cytokines or serum of HBV-ACLF patients. **(A, D)** The naïve CD4^+^ T cells of HC subjects (n = 6) were stimulated by the RPMI complete medium as control (CTR), dynabeads^®^ human T-activator CD3/CD28, IL-12 (10 ng/ml), IL-21 (20 ng/ml), IL-12+IL-21 and IL-17 (10 ng/ml) for 72 hours *in vitro*, and the frequencies of CD4^+^CXCR5^+^ICOS^+^ Tfh cells were demonstrated by flow cytometry, respectively. **(B, E)** The naïve CD4^+^ T cells of HC subjects were stimulated by CTR, dynabeads^®^ human T-activator CD3/CD28, serum from HC subjects (n = 6), and serum from HBV-ACLF patients for 72 hours *in vitro*, and the frequencies of CD4^+^CXCR5^+^ICOS^+^ Tfh cells were demonstrated by flow cytometry, respectively. **(C, F)** The naïve CD4^+^ T cells of HC subjects were stimulated by CTR, dynabeads^®^ human T-activator CD3/CD28, serum from HBV-ACLF patients (1:8) with or without IL-12 (1 µg/ml), IL-21 (1 µg/ml), IL-12/21 and IL-17(1 µg/ml) antibody (the antibody experiments were performed in the presence of 1:8 HBV-ACLF serum), for 72 hours *in vitro*, and the frequencies of CD4^+^CXCR5^+^ICOS^+^ Tfh cells were demonstrated by flow cytometry, respectively. Representative data of independent experiments are shown as median (range). *p < 0.05, **p < 0.01.

The possibility of soluble factors in the serum of HBV-ACLF patients inducing naïve CD4^+^ T cell differentiation to Tfh cells was investigated. CD4^+^CXCR5^+^ICOS^+^ Tfh cells were mildly induced by stimulation dynabeads^®^ human T-activator CD3/CD28, and there was a statistically significant increase after the addition of exogenous IL-12 and/or IL-21 (**Figures 4A, D**). This data is in line with findings that IL-12 and IL-21 are important in differentiation and generation of Tfh cells (19–21). As expected, exogenous IL-17 has no effect on differentiation of Tfh cells (**Figures 4A, D**). In our further study, naïve CD4^+^ T cells differentiated into Tfh cells in the presence of the stimulation dynabeads^®^ human T-activator CD3/CD28, HC subjects’ serum (n = 6) and HBV-ACLF patients’ serum (n = 7) with the RPMI complete medium. And the HBV-ACLF patients’ serum showed the strongest ability to induce Tfh differentiation (**Figures 4B, E**). Our preliminary data showed the best ratio of HBV-ACLF patients’ serum and the complete medium for Tfh cells induction was 1: 8, and then the effect reduced gradually as the ration decreased to 1: 64 (**Figures 4B, E**).

To confirm that the effect of the HBV-ACLF patients’ serum is due to IL-12 and/or IL-21, neutralizing antibodies to IL-12 or IL-21 were applied. The differentiation of Tfh cells was significantly decreased by addition of neutralizing antibody to IL-12 (*p* < 0.01), IL-21 (*p* < 0.01) or a combination of both (*p* < 0.01) for 72 hours (**Figures 4C, F**), which were ~65%, 63%, and 63% less, respectively. No add-on effect was observed in the presence of anti-IL-12 and IL-21 antibodies, compared to single anti-IL-12 or anti-IL-21 antibody alone. As expected, there was also no blocking effect of differentiation of Tfh cells in the presence of anti-IL-17 antibody (**Figures 4C, F**), despite an increased IL-17 level in HBV-ACLF patients’ serum.

**Figure 5:**
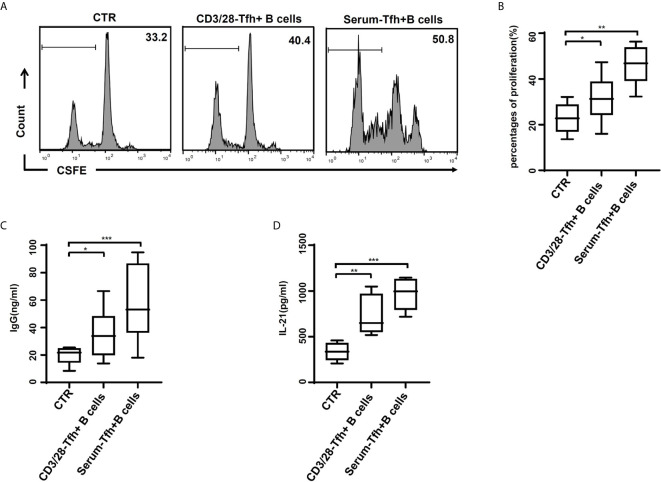
Tfh cells after stimulation of HBV-ACLF patients’ serum induced the proliferation and IgG production of B cells *in vitro*. **(A, B)** Naïve CD4^+^ T cells stimulated with the RPMI complete medium (CTR), with or without dynabeads^®^ human T-activator CD3/CD28 and HBV-ACLF patients’ serum, were cultured with naïve B cells in the presence of a surperantigen. The proliferation of CD19^+^ B cell in the CFSE dilution assay was evaluated quantitatively by comparing the percentages of cells that underwent cell division at least once. **(C, D)** IgG production **(C)** and IL-21 release **(D)** were dosed in the culture supernatants through ELISA. Representative data of independent experiments are shown as median (range). *p < 0.05, **p < 0.01, ***p < 0.001.

Moreover, the function of CD4^+^CXCR5^+^ICOS^+^ Tfh cells was explored. The proliferation of CD19^+^ B cells was significantly increased in CD19^+^ B cells cultures including naïve CD4^+^ T cells after stimulation with patients’ serum, compared to those with anti-CD3/28 or blank control (**Figures 5A, B**). The IgG level had similar pattern (**Figure 5C**). Consistently, the IL-21 levels were also increased in cultures including naïve CD4^+^ T cells (**Figure 5D**).

Thus, our data demonstrate that IL-12 and IL-21 are key factors in the HBV-ACLF patients’ serum for Tfh cell differentiation, which may participant in the disease progression and/or response to medical treatment.

## Discussion

Since the first report of HBV-ACLF, research into the pathogenesis of the disease has been unceasing. In our current study, CD4^+^CXCR5^+^ Tfh cells were identified in peripheral blood and liver tissues from a cohort of HBV-ACLF patients. The frequency of CD4^+^CXCR5^+^ Tfh cells was increased in PBMCs and liver tissue, consistent with MELD score in HBV-ACLF patients, compared to CHB and HC subjects. Furthermore, CD4^+^CXCR5^+^ICOS^+^ and CD4^+^CXCR5^+^IL-21^+^ Tfh cell subsets were increased in HBV-ACLF patients. The frequency of CD4^+^CXCR5^+^PD-1^+^ Tfh cell subsets showed no difference compared with other groups. These findings suggest that different Tfh cell subsets may have different regulation mechanisms in the initiation and progression of HBV-ACLF.

The finding of a significantly reduced frequency of Tfh cells in ameliorated, but not in non-ameliorated HBV-ACLF patients, supports the conclusion that Tfh cells play an important role in the development of HBV-ACLF. Therefore, the frequency of Tfh cells might be used as a novel predictor for the prognosis of HBV-ACLF. In fact, a recent study showed that TLR8 agonist GS-9688 (selgantolimod) promoted Tfh differentiation and function by increasing the expression of ICOS, which may be able to activate antiviral effector function (22). Thus, we suppose Tfh cells may be a therapeutic target in those treatment-resistant HBV-ACLF patients through regulating the function of Tfh cells in the future. The increased frequency of CD4^+^CXCR5^+^ Tfh cells was also observed in CHB patients, even though the pathogenesis of CHB and HBV-ACLF is completely different (23).

Tfh cells are mainly located in lymphoid follicles (6). Co-localization of CD4 and CXCR5 in the liver from HBV-ACLF patients suggests these double positive cells are Tfh cells (6). The correlation between CD4^+^CXCR5^+^ICOS^+^ Tfh cells and CD19^+^ B cells in HBV-ACLF patients is in accordance with other studies, which showed that Tfh cells regulate terminal differentiation of B cells (24, 25). Meanwhile, Tfh cells after stimulation of HBV-ACLF patients’ serum induced the proliferation and IgG production of B cells *in vitro*. Significantly up-regulated serum levels of IgG and IgM in HBV-ACLF patients invite speculation that humoral immunity also contributes to the pathogenesis of HBV-ACLF, which may be regulated by Tfh cells during the development of HBV-ACLF. However, the specificity of IgG and IgM remains unclear.

IL-12 plays a key role in enhancing Tfh cell differentiation in our current study, consistent with others (10). Our findings showed substantially elevated (~2-fold) IL-12 in the serum from HBV-ACLF patients compared to the other three groups, which is consistent with other study (26). IL-12 is mainly secreted by macrophages, and IL-12 secretion by macrophages is a seminal event in the initiation of the interferon-gamma (IFNγ) immune-activating cytokine cascade. Therefore, IL-12 seems to be secreted at the beginning of HBV-ACLF. It may not be significantly related to the progression of the disease (evaluated by MELD score, AST/ALT). Up-regulated differentiation of Tfh cells by HBV-ACLF patients’ serum can be blocked by an anti-IL-12 neutralizing antibody *in vitro*, confirming the importance of IL-12 in differentiation of Tfh cells in the pathogenesis of HBV-ACLF. Considering the discrepancy between IL-12’s crucial role *in vitro* and its irrelevance to pathological markers, we suppose serum IL-12 level may be associated with the total number of CD4^+^CXCR5^+^ Tfh cells, rather than the specific subset, CD4^+^CXCR5^+^ICOS^+^ Tfh cells.

Our findings showed an increased serum IL-21 levels in HBV-ACLF patients compared to the other three groups. Hou and his colleagues found that serum IL-21 level and IL-21-secreting CD4^+^ Th cell frequency (mainly IL-21-secrating Th17 cells) increased in HBV-ACLF patients (27). Interestingly, Hou’s study mentioned that the expression of IL-21 in IL-17A^-^CD4^+^ cell subsets was much higher than that in IL-17A^+^CD4^+^ cell subsets. In this case, we suppose there might exist other CD4^+^ cell subsets secreting IL-21 in HBV-ACLF. And in our study, CD4^+^CXCR5^+^ Tfh cells increased in HBV-ACLF were found to be another IL-21-secreting T helper cell subset. Meanwhile, IL-21 is another important cytokine in the development of Tfh cells, which acts in both a paracrine and autocrine fashion (12, 28). IL-21 facilitates an effective immune response in acute HBV infection *via* enhancement of B cell terminal differentiation (13). IL-21 level was up-regulated by ~5-fold in the serum of HBV-ACLF patients. The IL-21 enriched HBV-ACLF patients’ serum was found to induce naïve CD4^+^ T cells differentiate to Tfh cells, in turn blocked by neutralizing anti IL-21 antibody, supporting the key role of IL-21 in the differentiation of Tfh cells during the development of HBV-ACLF. There is a significant correlation between IL-21 production and Tfh number, clinical score, liver function (ALT/AST), further confirming the importance of IL-21 in the generation of Tfh cells during the development of HBV-ACLF.

Interestingly, there is no add-on effect of IL-12 and IL-21 on Tfh differentiation from naïve CD4^+^ T cells, nor did anti IL-12 and IL-21 neutralizing antibodies. This finding might be due to IL-12 inducing IL-21 *via* the transcription factors STAT1, STAT3, STAT4 and STAT5 (29), whereas IL-21 uses only the STAT3 pathway (30). This may further support the hypothesis that IL-12 has a greater effect on Tfh cell development than that of IL-21.

Other studies showed that IL-17 exacerbates liver damage in liver injury (31), but the role of IL-17 in the development of Tfh cells was unclear. We found no effective role for IL-17 in the differentiation of naïve CD4^+^ T cells into Tfh cells *in vitro*, despite highly elevated IL-17 level in HBV-ACLF patients’ serum. Furthermore, anti-IL-17 neutralizing antibody didn’t modify the differentiation of Tfh cells in the HBV-ACLF patients’ serum. This data suggests that IL-17 doesn’t affect the development of Tfh cells in HBV-ACLF patients.

Systemic inflammatory response, including TNF, IL-8, IL-6, and IL-2, contributes to the transition from stable chronic liver disease to HBV-ACLF, which is associated with morbidity and mortality of hepatitis (32). These pro-inflammatory cytokines were up-regulated significantly in HBV-ACLF, consistent with both local and systemic acute inflammation during the development of HBV-ACLF. This increase in pro-inflammatory cytokines may explain our observation of an increase in the anti-inflammatory cytokine IL-4.

There was no significant change in IL-1β, IFN-γ, IL-10 or TGF-β in the HBV-ACLF patients’ serum compared to that of other three groups. This data suggests a different pathogenesis of HBV-ACLF compared to other liver conditions, a point which will be clarified in our future studies of the underlying mechanism.

In summary, up-regulated Tfh cells and IL-21 are well correlated with severity/amelioration of HBV-ACLF, and up-regulated IL-12 may be correlated with disease initiation. IL-12/21 enriched HBV-ACLF patients’ serum enhanced Tfh cells differentiation from naïve CD4^+^ T cells, an interaction blocked by neutralizing anti IL-12/21 antibodies. These data suggest there is a close relationship between IL-12/21, Tfh cells and disease progression in HBV-ACLF patients. This information provides insights into the pathogenesis of HBV-ACLF, and stands to profoundly impact on the development of novel strategies for the treatment of HBV-ACLF.

## Data Availability Statement

The original contributions presented in the study are included in the article/**Supplementary Material**. Further inquiries can be directed to the corresponding authors.

## Ethics Statement

The studies involving human participants were reviewed and approved by Ethics Committee of Ruijin Hospital, Shanghai Jiaotong University School of Medicine. The patients/participants provided their written informed consent to participate in this study.

## Author Contributions

BD wrote the original draft. JT and RY were involved in the data curation. YT and TJ performed validation. WC and YD wrote review and editing. All authors contributed to the article and approved the submitted version.

## Funding

This study was funded by the Natural Science Foundation of Shanghai (20ZR1433500), the Major Project of National Thirteenth Five-year Plan (2017ZX09304016), and the Shanghai Municipal Key Clinical Specialty (shslczdzk1103).

## Conflict of Interest

The authors declare that the research was conducted in the absence of any commercial or financial relationships that could be construed as a potential conflict of interest.

## Supplementary Material

Supplementary Figure 1
**(A)** Lymphocytes were gated according to FSC and SSC in human PBMCs, and CD4^+^ T cells were gated in lymphocytes (66.7% lymphocytes in PBMCs, and 35.0% CD4^+^ T cells in lymphocytes). **(B)** The frequencies of CD4^+^CXCR5^+^PD-1^+^ Tfh cells, CD4^+^CXCR5^-^ICOS^+^ T cells and CD4^+^CXCR5^+^ICOS^+^PD-1^+^ Tfh cells in the PBMCs from HBV-ACLF (n = 36), M-CHB (n = 21), S-CHB (n = 32) patients and HC subjects (n = 10) were demonstrated by flow cytometry. **(C)** The frequencies of CD4^+^CXCR5^+^PD-1^+^ Tfh cells, CD4^+^CXCR5^-^ICOS^+^ T cells and CD4^+^CXCR5^+^ICOS^+^PD-1^+^ Tfh cells in the PBMCs from HBV-ACLF, M-CHB, S-CHB patients, HC subjects were analyzed using Mann-Whitney U test. Representative data of independent experiments are shown as median (range).
