# Addendum: Increased Circulating T Follicular Helper Cells Induced *via* IL-12/21 in Patients With Acute on Chronic Hepatitis B Liver Failure

**DOI:** 10.3389/fimmu.2022.798058

**Published:** 2022-03-11

**Authors:** Bingying Du, Jiaming Teng, Rongkun Yin, Yuanyuan Tian, Tingwang Jiang, Yanan Du, Wei Cai

**Affiliations:** ^1^ Department of Infectious Diseases, Ruijin Hospital, Shanghai Jiao Tong University School of Medicine, Shanghai, China; ^2^ Department of Infectious Diseases, Tongren Hospital, Shanghai Jiao Tong University School of Medicine, Shanghai, China; ^3^ Department of Hematology, Children Hospital, Soochow University, Suzhou, China; ^4^ Clinical Research Centre, The Affiliated Changshu Hospital of Xuzhou Medical University, Changshu, China

**Keywords:** T follicular helper cell, hepatitis B virus-related acute on chronic liver failure, pathogenesis, interleukin-21, interleukin-12

In response to comments on PubPeer, the author uploaded the raw data as **Supplementary Material 2** to support Figures 4A–C in the original file. The authors apologize to the readership for any inconvenience caused.

These experiments were completed from July to September 2012. The workspace can’t be used due to the upgrade of the version of FlowJo. The data were reanalyzed using the same gating and labelling in FlowJo version 10.6. The results are presented in the format of Pseudocolour. The trend of the data has not changed, which does not affect the original results.

The authors uploaded the FCS format raw data and Pseudocolour format graphical of all 6 patients, which mentioned in the original article (n=6, and original figure 4 shows the flow analysis data of patient #1).

The documents are as follows:

1) Data 1: FACS raw files of patient (n=6. A patient’s data is summarized in a folder. Each folder contains 23 FCS files, which are marked according to the processing.)

2) Data:2: Figure panels of Pseudocolour format graphical (n=6. One page per patient) in PPT.

## Publisher’s Note

All claims expressed in this article are solely those of the authors and do not necessarily represent those of their affiliated organizations, or those of the publisher, the editors and the reviewers. Any product that may be evaluated in this article, or claim that may be made by its manufacturer, is not guaranteed or endorsed by the publisher.

