# Supplementary figures and images for: Increased Circulating T Follicular Helper Cells Induced via IL-12/21 in Patients With Acute on Chronic Hepatitis B Liver Failure
Source: Front Immunol. 2021 Mar 31;12:641362. doi: 10.3389/fimmu.2021.641362 (PMC8044369; doi:10.3389/fimmu.2021.641362)

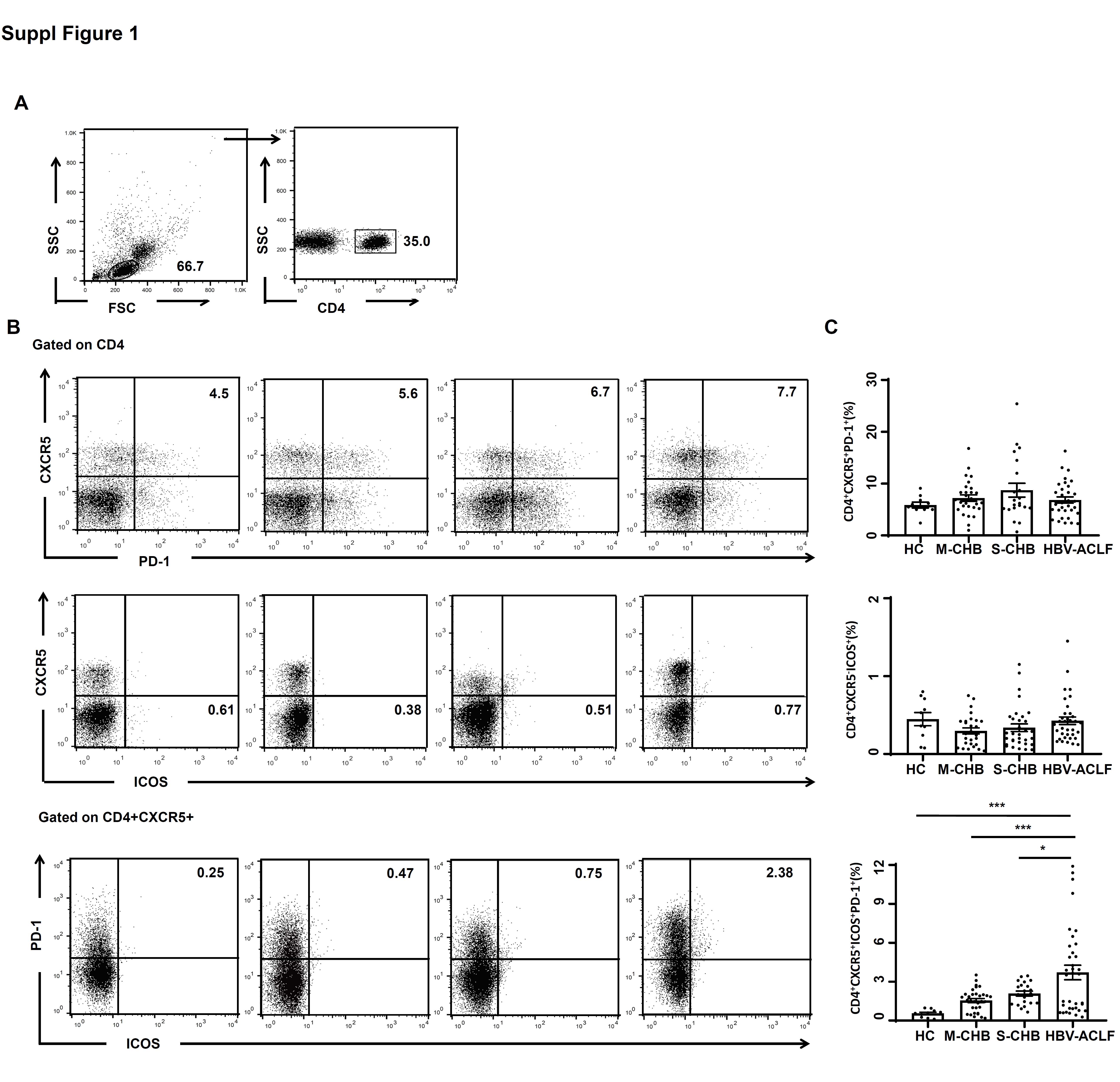

Supplement: Supplementary Figure 1 — (A) Lymphocytes were gated according to FSC and SSC in human PBMCs, and CD4+ T cells were gated in lymphocytes (66.7% lymphocytes in PBMCs, and 35.0% CD4+ T cells in lymphocytes). (B) The frequencies of CD4+CXCR5+PD-1+ Tfh cells, CD4+CXCR5-ICOS+ T cells and CD4+CXCR5+ICOS+PD-1+ Tfh cells in the PBMCs from HBV-ACLF (n = 36), M-CHB (n = 21), S-CHB (n = 32) patients and HC subjects (n = 10) were demonstrated by flow cytometry. (C) The frequencies of CD4+CXCR5+PD-1+ Tfh cells, CD4+CXCR5-ICOS+ T cells and CD4+CXCR5+ICOS+PD-1+ Tfh cells in the PBMCs from HBV-ACLF, M-CHB, S-CHB patients, HC subjects were analyzed using Mann-Whitney U test. Representative data of independent experiments are shown as median (range). [file Image_1.jpeg]
