# Supplementary material for: Addendum: Increased Circulating T Follicular Helper Cells Induced via IL-12/21 in Patients With Acute on Chronic Hepatitis B Liver Failure
Source: Front Immunol. 2022 Mar 11;13:798058. doi: 10.3389/fimmu.2022.798058 (PMC8962647; doi:10.3389/fimmu.2022.798058)
Supplement: Supplementary file 7 [file DataSheet_7.zip › Data 2.pptx]

## Slide 1
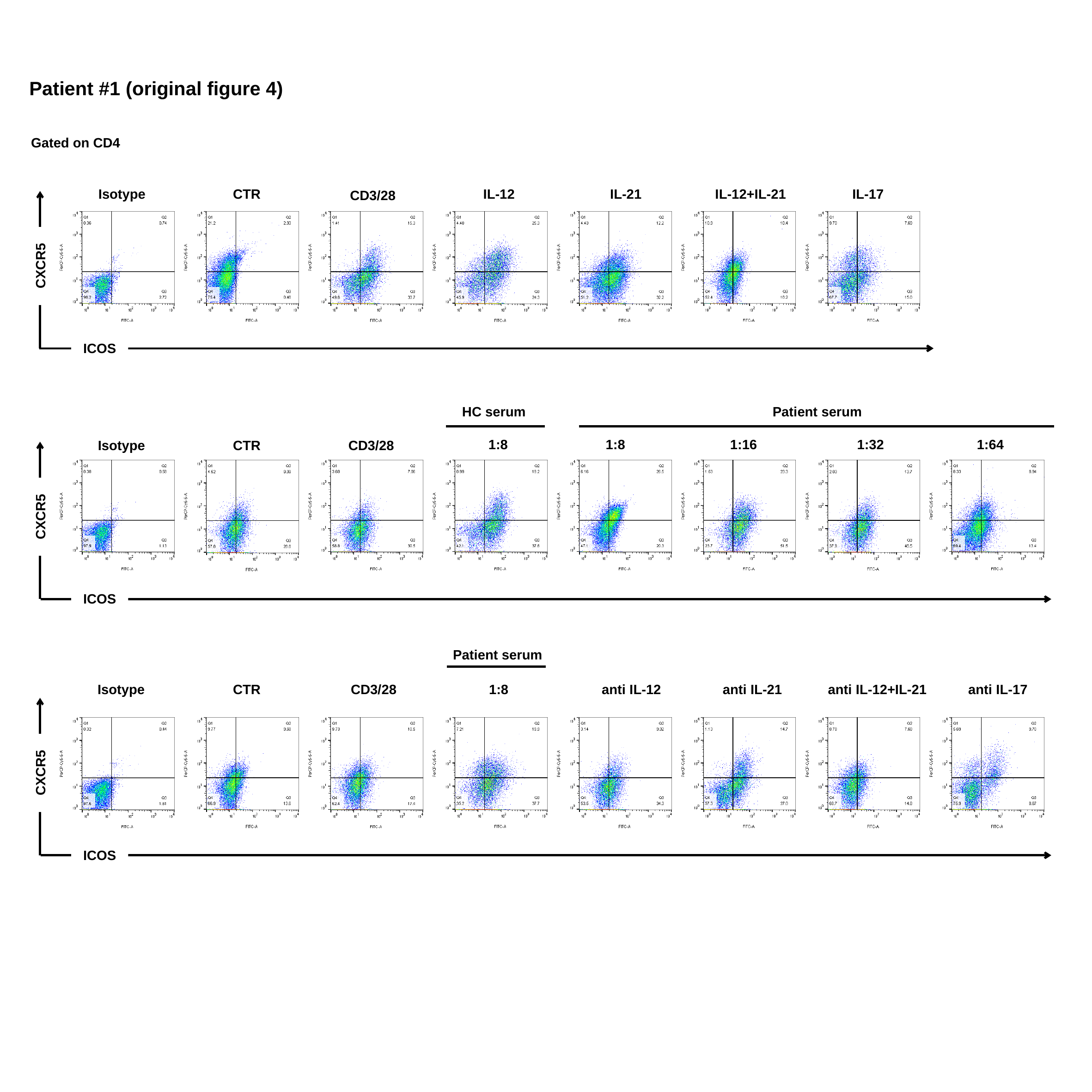

Patient #1 (original figure 4)
Gated on CD4
Isotype
CTR
IL-12
IL-21
IL-12+IL-21
IL-17
CD3/28
CXCR5
ICOS
HC serum
Patient serum
1:8
1:8
1:16
1:32
1:64
Isotype
CTR
CD3/28
CXCR5
ICOS
Patient serum
Isotype
CTR
CD3/28
1:8
 anti IL-12
anti IL-21
anti IL-12+IL-21
anti IL-17
CXCR5
ICOS

## Slide 2
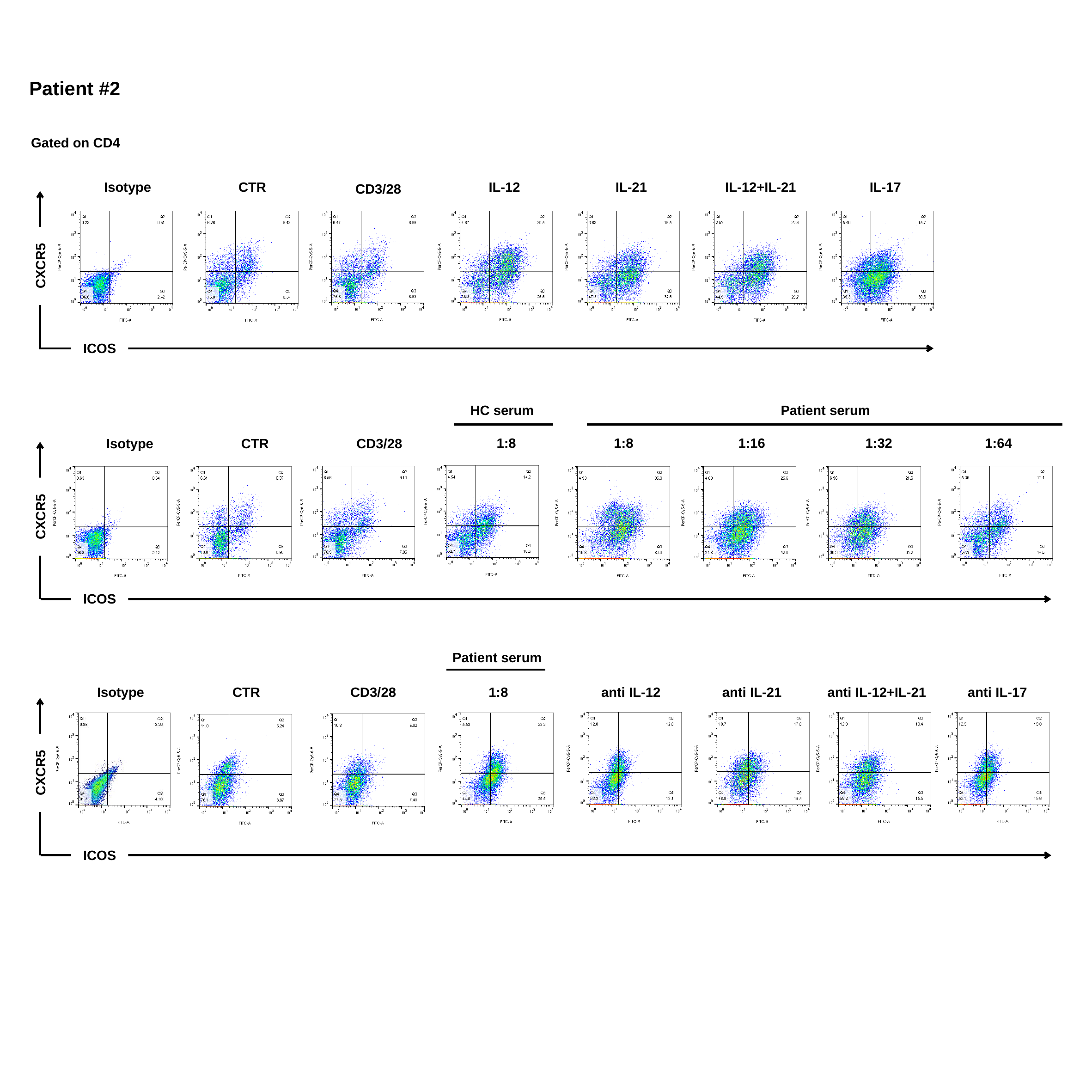

Patient #2
Gated on CD4
Isotype
CTR
IL-12
IL-21
IL-12+IL-21
IL-17
CD3/28
CXCR5
ICOS
HC serum
Patient serum
1:8
1:8
1:16
1:32
1:64
Isotype
CTR
CD3/28
CXCR5
ICOS
Patient serum
Isotype
CTR
CD3/28
1:8
 anti IL-12
anti IL-21
anti IL-12+IL-21
anti IL-17
CXCR5
ICOS

## Slide 3
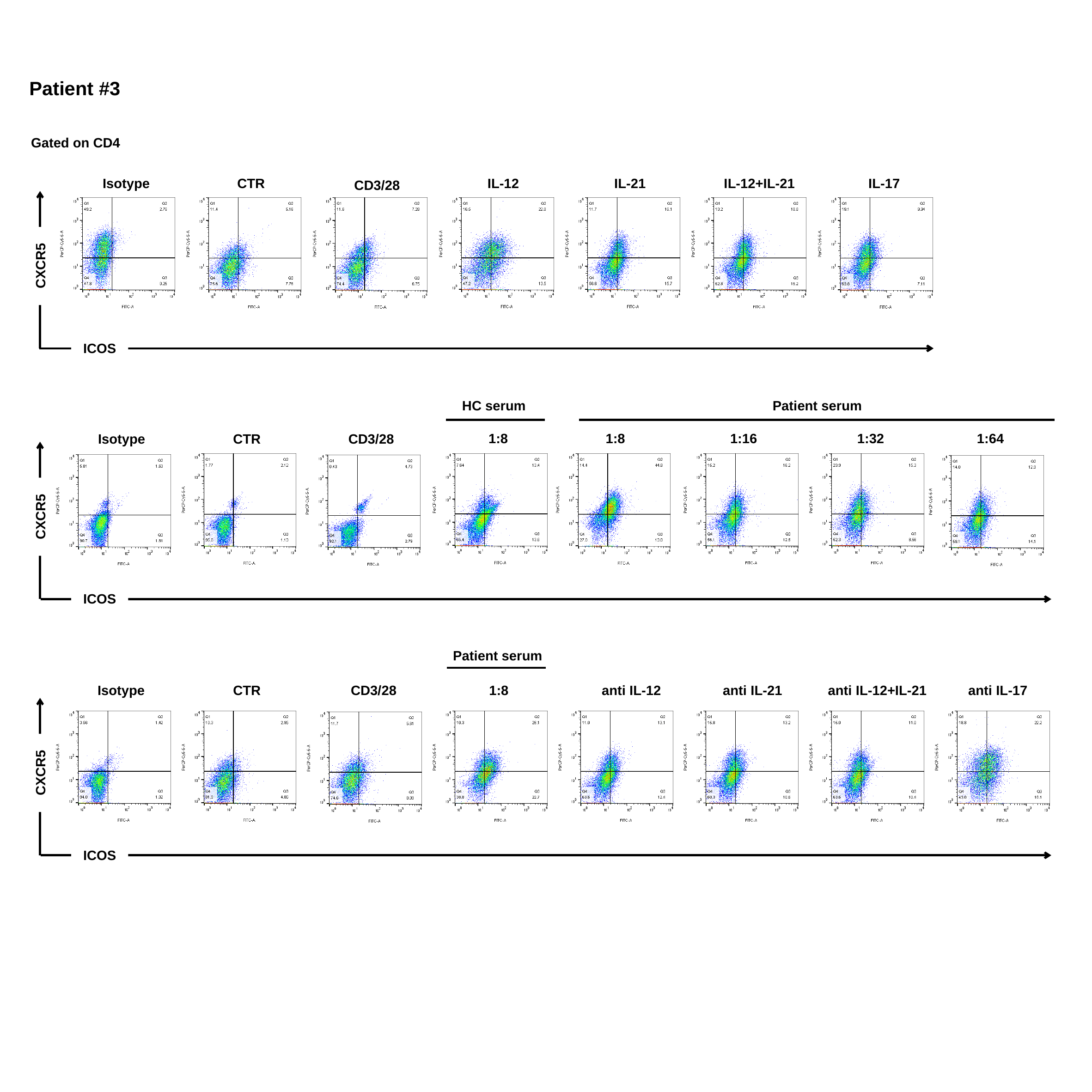

Patient #3
Gated on CD4
Isotype
CTR
IL-12
IL-21
IL-12+IL-21
IL-17
CD3/28
CXCR5
ICOS
HC serum
Patient serum
1:8
1:8
1:16
1:32
1:64
Isotype
CTR
CD3/28
CXCR5
ICOS
Patient serum
Isotype
CTR
CD3/28
1:8
 anti IL-12
anti IL-21
anti IL-12+IL-21
anti IL-17
CXCR5
ICOS

## Slide 4
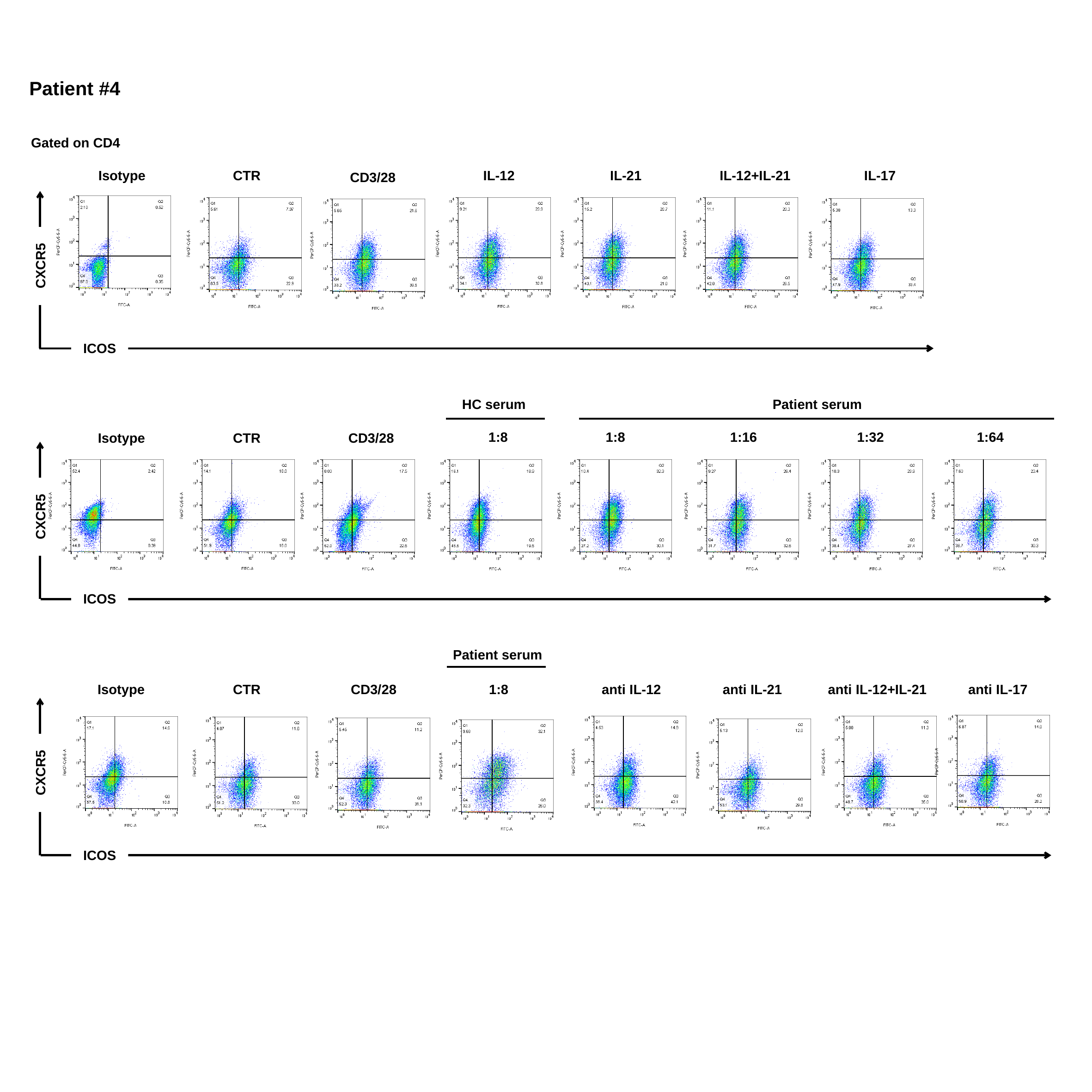

Patient #4
Gated on CD4
Isotype
CTR
IL-12
IL-21
IL-12+IL-21
IL-17
CD3/28
CXCR5
ICOS
HC serum
Patient serum
1:8
1:8
1:16
1:32
1:64
Isotype
CTR
CD3/28
CXCR5
ICOS
Patient serum
Isotype
CTR
CD3/28
1:8
 anti IL-12
anti IL-21
anti IL-12+IL-21
anti IL-17
CXCR5
ICOS

## Slide 5
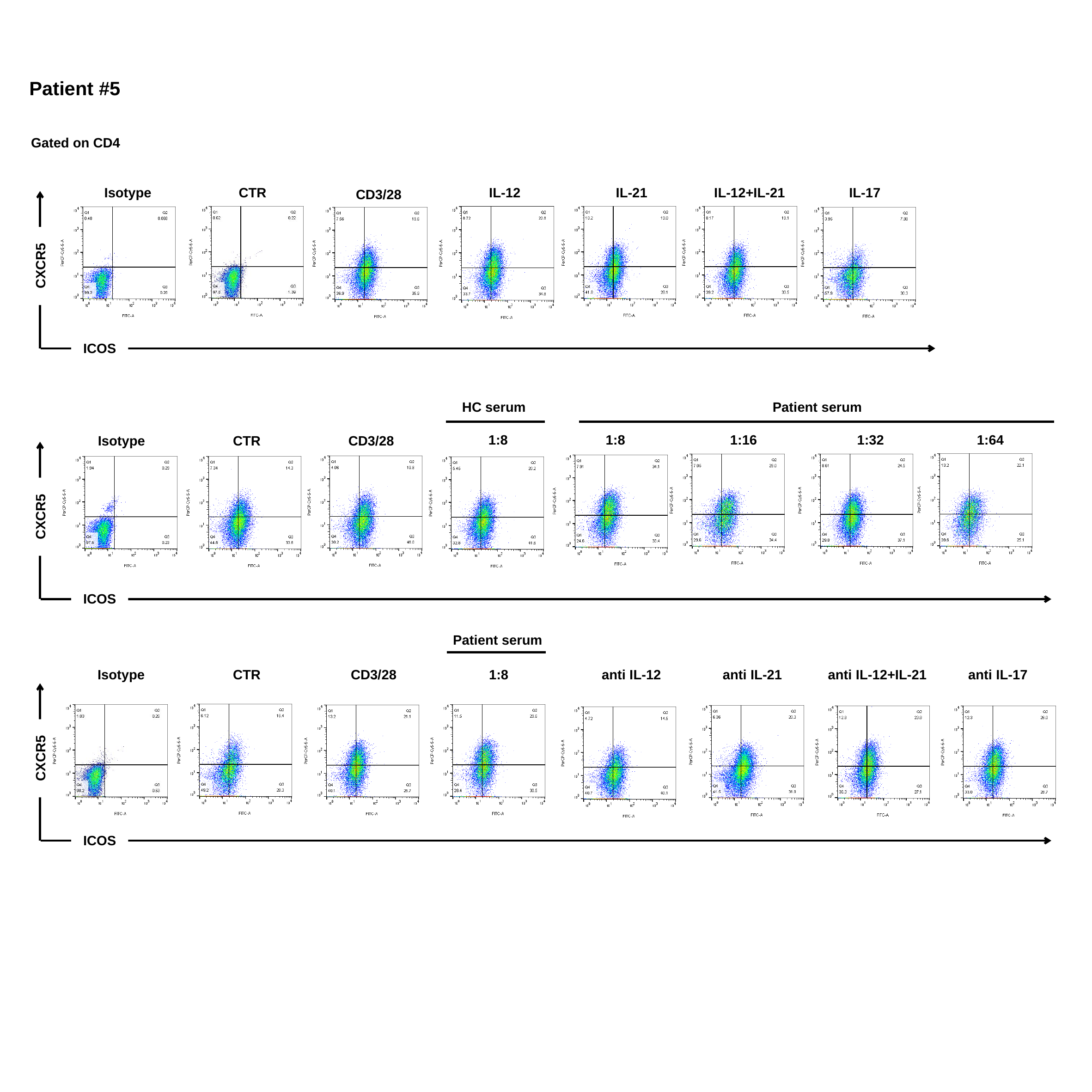

Patient #5
Gated on CD4
Isotype
CTR
IL-12
IL-21
IL-12+IL-21
IL-17
CD3/28
CXCR5
ICOS
HC serum
Patient serum
1:8
1:8
1:16
1:32
1:64
Isotype
CTR
CD3/28
CXCR5
ICOS
Patient serum
Isotype
CTR
CD3/28
1:8
 anti IL-12
anti IL-21
anti IL-12+IL-21
anti IL-17
CXCR5
ICOS

## Slide 6
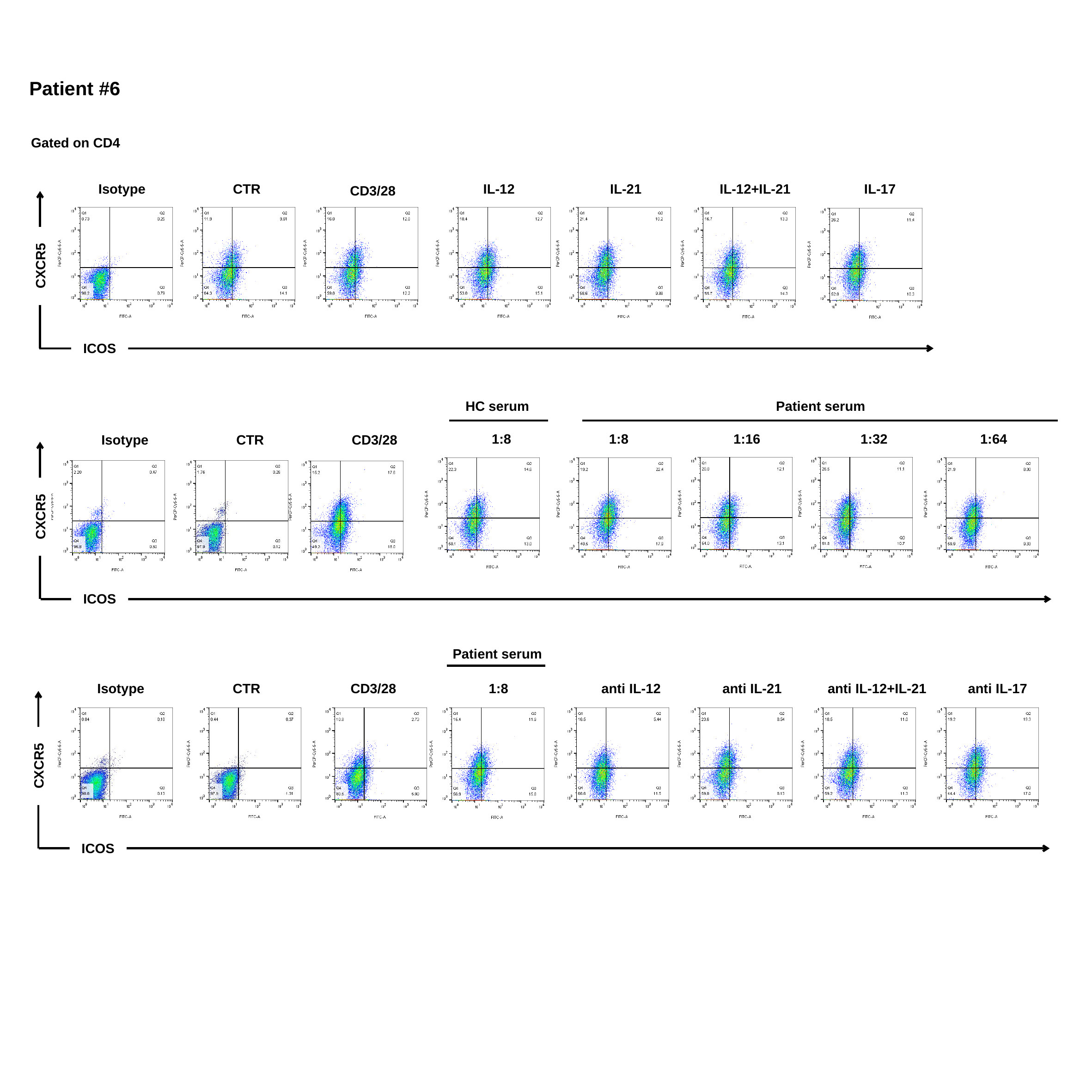

Patient #6
Gated on CD4
Isotype
CTR
IL-12
IL-21
IL-12+IL-21
IL-17
CD3/28
CXCR5
ICOS
HC serum
Patient serum
1:8
1:8
1:16
1:32
1:64
Isotype
CTR
CD3/28
CXCR5
ICOS
Patient serum
Isotype
CTR
CD3/28
1:8
 anti IL-12
anti IL-21
anti IL-12+IL-21
anti IL-17
CXCR5
ICOS
